# Cell Type-Specific Separation of Subicular Principal Neurons during Network Activities

**DOI:** 10.1371/journal.pone.0123636

**Published:** 2015-04-14

**Authors:** Joanna Eller, Shota Zarnadze, Peter Bäuerle, Tamar Dugladze, Tengis Gloveli

**Affiliations:** 1 Cellular and Network Physiology Group, Institute of Neurophysiology, Charité-Universitätsmedizin Berlin, Berlin, Germany; 2 Cluster of excellence “NeuroCure”, Berlin, Germany; 3 Bernstein Center for Computational Neuroscience Berlin, Berlin, Germany; Consejo Superior de Investigaciones Cientificas—Instituto Cajal, SPAIN

## Abstract

The hippocampal output structure, the subiculum, expresses two major memory relevant network rhythms, sharp wave ripple and gamma frequency oscillations. To this date, it remains unclear how the two distinct types of subicular principal cells, intrinsically bursting and regular spiking neurons, participate in these two network rhythms. Using concomitant local field potential and intracellular recordings in an *in vitro* mouse model that allows the investigation of both network rhythms, we found a cell type-specific segregation of principal neurons into participating intrinsically bursting and non-participating regular spiking cells. However, if regular spiking cells were kept at a more depolarized level, they did participate in a specific manner, suggesting a potential bimodal working model dependent on the level of excitation. Furthermore, intrinsically bursting and regular spiking cells exhibited divergent intrinsic membrane and synaptic properties in the active network. Thus, our results suggest a cell-type-specific segregation of principal cells into two separate groups during network activities, supporting the idea of two parallel streams of information processing within the subiculum.

## Introduction

The subiculum constitutes the major output structure of the hippocampal formation [1]. As the final relay in a polysynaptic loop between the entorhinal cortex (EC) and the hippocampus, it integrates and distributes processed spatial and mnemonic information to cortical and subcortical brain regions [2–4]. Within the subiculum there are two types of principal cells (PCs) that can be discriminated based on their firing properties: regular spiking (RS) and intrinsically bursting (IB) neurons [5–10]. Bursting has been functionally associated with an amplification of neuronal signals by increasing the efficiency of cellular communication [11,12]; accordingly, the subicular IB cell appears to be the most promising cell type regarding the involvement in network activity [9,13,14]. Furthermore, subicular IB and RS cells were shown to target different brain regions, the presubiculum and EC, respectively [15,16]. The distinct target areas, differential intrinsic and firing properties as well as the different reactions to neurotransmitters point towards specific functional tasks of the two cell types within the hippocampal information processing [17–21].

Network oscillations in the theta (4–10 Hz) and gamma (30–90 Hz) frequency range as well as sharp waves (SPW), usually superimposed by ripple oscillations (100–250 Hz), are the most prominent activity pattern within the hippocampal formation. Sharp wave-ripples are associated with memory consolidation whereas the integration of new information takes place during gamma oscillations, thus these two oscillatory states appear to be functionally connected [22–26]. It has been shown that the subiculum is capable of generating SPWs intrinsically with the subicular PCs displaying a mixed excitatory and inhibitory current [14]. Furthermore, early studies have also explored subicular network activity in the gamma frequency range after tetanic stimulation of the hippocampal CA1 area and the subiculum itself [27,28]. In addition, in the whole hippocampal preparation, it has been suggested that the subiculum is capable of generating gamma frequency oscillations intrinsically and spontaneously [29]. However, the distinct involvement of the subicular IB and RS cells in the two major network rhythms remains so far unexplored.

Using simultaneous local field potential (LFP) and sharp microelectrode recordings in an *in vitro* acute hippocampal slice preparation that permits the reproduction of the two prominent network rhythms, SPW and gamma frequency oscillations, we investigated the intrinsic and synaptic properties of subicular PCs as well as their functional involvement in both network rhythms. We found a prominent segregation of PCs into two main groups, participating IB cells and RS neurons that are predominantly silent but potentially capable of reinforcing the network state. Our results indicate two separate ways of information processing represented by the two distinct subicular PC classes. In this context, we suggest a bimodal working model that depends on the functional state of the subicular PC types: a low or moderate activity level seems to result in an active participation of the IB cell type whereas a high state of excitation appears to be necessary to sufficiently arouse the RS cell type. Together with divergent synaptic properties during the network activities, our results support the idea of two independent streams of information flow within the hippocampal output structure.

## Materials and Methods

### Ethics statement

All procedures were approved by the Regional Berlin Animal Ethics Committee, T 0124/05, and are in full compliance with national regulations.

### Slice preparation

Experiments were performed on adult (>6 weeks of age) C57/Bl6 mice of both sex. The animals were anesthetized with inhaled isoflurane and decapitated. Horizontal, 400 μm thick slices were obtained and placed in an ‘interface’ chamber at 34 ± 1°C. Minislices were created by isolating the subiculum via microscissors through cuts around its perimeters. For minislice preparation and subsequent recordings horizontal slices from all levels (ventral, middle, and dorsal) were used, while intracellular recordings were performed only in slices from the middle level of the subiculum. Additionally, 400 μm thick sagittal slices were prepared in order to evaluate a possible dependency of gamma generation on the slice orientation [30]. Slice preparations were illustrated by sketches based on the mouse brain atlas [31]. Prior to recordings the slices were allowed to rest for at least 1 hour for recovery. The solution used during preparation, incubation, and recordings was made with deionized distilled water and contained (in mM): NaCl, 129; KCl, 3; NaH_2_PO_4_, 1.25; CaCl_2_, 1.6; MgSO_4_, 1.8; NaHCO_3_, 21; glucose, 10; saturated with 95% O_2_ and 5% CO_2_.

### Extracellular and intracellular recordings

Concomitant extracellular LFP and intracellular recordings were obtained from the subicular stratum pyramidale of the middle horizontal slice preparations. Kainic acid (400 nM) was applied to the bath to induce network oscillations in the gamma frequency range. The LFP recordings in the intact and isolated subiculum were amplified, digitized with a sampling rate of 10 kHz (Digidata 1322A, Axon Instruments) and analyzed with the pClamp software package (notch filter 50 Hz, butterworth filter 2–2000 Hz; Axon Instruments). Oscillatory peak power and frequency was determined by fast Fourier transform (FFT) using a 0.5 Hz spectral resolution.

Sharp microelectrode recordings were obtained simultaneously to LFP recordings from the pyramidal cells in the principal cell layer of the subiculum. The intracellular solution contained 2 M potassium acetate. The tip resistance ranged from 60–110 MΩ. Cells were impaled and then allowed to rest for 10 minutes. Only cells with a resting membrane potential (RMP) ≤ −50 mV and an action potential (AP) half width of ≤1.2 ms were accepted for further measurements. Signals were amplified by an IR-183 intracellular recording amplifier (Neuro Data Instruments Corp.) with an active bridge circuit and digitalized using a 16 Bit Data Acquisition system (Digidata 1322A, Axon Instruments). The bridge balance was monitored continuously and adjusted as needed. The transient suppression was used to remove the capacitance artifacts in some of the recordings. Excitatory postsynaptic potentials (EPSP) and inhibitory postsynaptic potentials (IPSP) were recorded at −80 mV and 0 mV, respectively, to analyze these events in the absence of corresponding receptor antagonists. The simultaneous recorded LFP was digitalized with a sampling rate of 2 kHz (Digidata 1440A, Axon Instruments) and analyzed with the pClamp software package (notch filter 50 Hz, butterworth filter 2–100 Hz; Axon Instruments). Oscillatory peak power and frequency was determined by FFT with a 1 Hz spectral resolution.

The subicular PCs were classified as regular spiking (RS) or intrinsically bursting (IB) based on their firing properties through current injections as described previously [5–7,9,10]. If PCs did not fire any APs at resting membrane potential (RMP) during a one minute period of spontaneous sharp wave (SPW) or gamma network oscillation they were further categorized as silent (or otherwise as active) with respect to the corresponding network state.

### Data analysis

The RMP was measured from the voltage baseline without current injection after an initial impalement-induced depolarization had subsided. All measured values were corrected for an offset when the microelectrode was withdrawn from the cell. For the offset correction the tip potential within the cell was subtracted from the voltage potential measured in the extracellular space at the end of the experiment. Current injections in steps of 20 pA, starting at −320 pA and increasing up to 200 pA with a step duration of 500 ms were applied to characterize the neuronal discharge behavior. The input resistance (R_in_) was determined from the cell’s response to a −100 pA current pulse calculated after any sag potential had subsided. The time constant (τ) represents the time taken to reach 63% of the steady-state voltage deflection during the same current pulse. During negative current injection the degree of hyperpolarization-activated sag potential was measured. The sag was defined as the maximum of voltage deflection (V_peak_) from a baseline of steady state voltage deflection (V_ss_) in response to a −100 pA, −200 pA, and −300 pA hyperpolarizing current injection, (100x (V_peak_ −V_ss_)/V_peak_; [18]). The AP threshold was determined by applying small steps of depolarizing or hyperpolarizing current injections. For inactive cells, the membrane potential that cause the cell to initiate AP firing was considered as AP threshold. Cells that fired spontaneous AP at RMP were hyperpolarized and the last membrane potential at which the cell was active was accepted as threshold. The afterdepolarization (ADP) and afterhyperpolarization (AHP) were defined as the corresponding maximum deflection. The accommodation of AP firing was determined by depolarizing current injection with a progressive increase of 40 pA per step, a step duration of 1 s and a maximum current injection of 500 pA. Due to the fact that high current injections can result in distorted AP waveforms accommodation was measured at the last current step displaying normal AP shapes. The interspike interval between the first (t_1_) and the last (t_2_) pair of APs was measured and the accommodation was calculated as the time difference between the first pair of APs divided by the time difference of the last pair of APs, (100x Δt_1_/Δt_2_). Initial AP bursts or salvos were discarded for accommodation analysis.

The recorded data were further processed in Matlab (The Mathworks, Inc.) with custom written routines. EPSP/IPSP recordings during gamma network oscillations were high pass filtered at 2 Hz with the pClamp software prior to the Matlab import. In Matlab 50 Hz noise was removed with a second-order IIR notch filter and LFP traces were zero-phased bandpass filtered with a butterworth filter from 3 to 100 Hz unless indicated otherwise. For illustrative purposes an FFT-based spectrogram (1 s time bins, hamming window, 50% overlap) was computed. The SPW-associated high frequency ripple component was not investigated systematically. For exemplary illustration of the ripple component a complex Morlet wavelet transform (cmor2-1) was used (bandpass filter 100–300 Hz). The mean frequency component of each ripple episode was defined as the spectral frequency analog with the maximal peak power of the wavelet transform. In order to analyze neural activity with respect to the prevalent oscillatory network pattern SPW and gamma frequency oscillatory peaks as well as APs and EPSPs/IPSPs were identified as maximal deflections above a manual set threshold. The maximum of LFP oscillatory peak deflections were defined as time 0 and the averaged time difference of the corresponding AP, EPSP or IPSP triggered peaks in the simultaneous intracellular recording trace was calculated. For SPW with a maximal negative LFP peak deflection the minimum was defined as time 0. However, the distribution of the data divided with respect to the two SPW polarities did not differ statistically significant. Therefore, data for all SWP analysis were pooled together, while for clarity reasons the illustrated SPW mean LFP signal is solely based on the positive waveform deflections. In dependency of the prevalent network pattern a mean peak EPSP and IPSP value was calculated for each recording. The mean peak EPSP and IPSP amplitude distribution of all analyzed recordings were further displayed as box plots and aggregated to a grand mean population value. Additionally, aggregated spike time points were displayed as a cumulative time histogram with a temporal resolution of 1 ms. PCs were considered to be phase-locked if the LFP triggered AP histogram displayed a clear peak of AP generation. A phase distribution of the phase-locked AP and IPSP mean values were calculated for each cell type and network state based on normalized oscillatory cycle length (24 ms for gamma frequency oscillations, 80 ms for SPWs, with the peak assigned to 180° each) and displayed as polar diagrams.

The paired student’s t-test was used for statistical analysis and the assumption of normal distribution was justified by the Lilliefors test. Differences were considered statistically significant if p < 0.05. Average values are expressed as mean ± SEM.

### Biocytin staining

2% Biocytin was added to the intracellular solution for staining. Slices were processed as described previously [32]. In brief, slices were immersed overnight in a fixative solution containing 4% paraformaldehyde in 0.1 M phosphate buffer (PB). They were then washed three times in 0.1 M PB. The avidin–biocytin complex reaction (Vectastain, ABC kit, Camon laboratory service) was allowed to occur overnight at 4°C in the presence of 0.3% Triton X-100 (Sigma-Aldrich). Afterwards the sections were rinsed several times before development with 0.02% diaminobenzidine in 0.1 M PB. The reaction product was intensified with 0.5% OsO_4_ and sections were mounted and coverslipped. Morphological data were examined by visual inspection and photomicrographs were taken. In order to achieve a depth-field view of the microscopic image for illustration purposes stack processing of consecutive focal planes were performed with the combine ZP software package (Alan Hadley, GNU public license).

## Results

### Network oscillations within the subiculum

**Fig 1:**
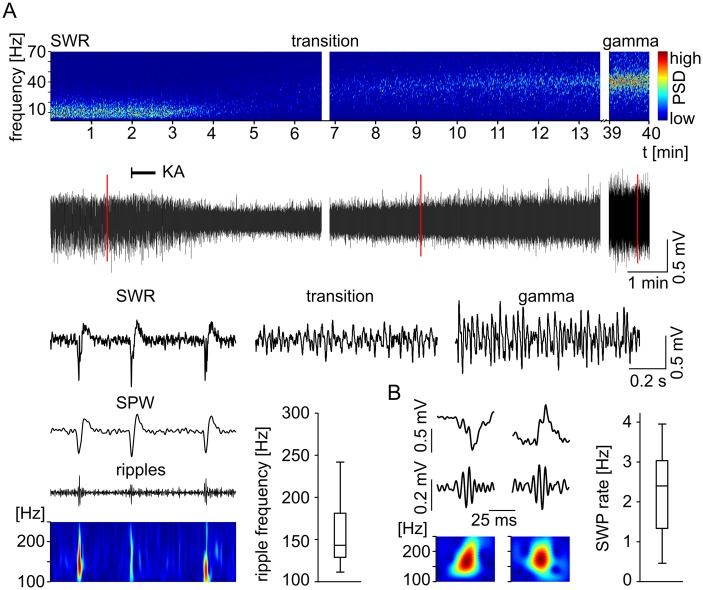
Sharp wave and gamma network oscillations within the subiculum. (A) Spectrogram (top) with color-coded power spectral density (PSD) exemplifies the transition from spontaneously occurring sharp wave-ripples (SWR) to gamma frequency oscillations within the subiculum. The corresponding LFP recordings are displayed below. The application of kainic acid (KA, onset is marked by black line) abolishes the SWR rhythm and induces, after a brief transitory state, a stable oscillatory gamma rhythm. The recording interruptions of the top spectrograms and the underlying LFP traces are 12 s (middle) and 25 min (right). Red lines mark three examples that are illustrated below with higher temporal resolution (SWR, transition, gamma). (A, bottom, left) The SWR (filtered 2–300 Hz), the corresponding SPW (2–50 Hz) and the ripple components (100–300 Hz) supplemented by the color-coded power spectral density wavelet transform. (A, bottom, right) The boxplot depicts the distribution of the wavelet peak power spectral frequencies of 100 analyzed consecutive ripple events of the upper example trace. (B) Sharp waves of both polarities are exemplified on the left with each SWR trace (2–300 Hz, top), the ripple trace (100–300 Hz, middle) and the corresponding wavelet transform as color-coded power spectral density plot (bottom). The boxplot (right) illustrates the distribution of the mean SWP rates of all slices investigated (n = 42).

Our *in vitro* model allowed us to investigate two major network activities within the subiculum, SPW and gamma frequency oscillations (Fig 1A). The SPWs occurred spontaneously (n = 42 slices) with a mean frequency of 2.2 Hz (Fig 1B). We detected two different groups of SPW distinguished by polarities, one displaying a positive maximum deflection (n = 31) and the other a negative one (n = 11, Fig 1B). Fast ripple oscillations (Fig 1A) superimposing the SPW component could be found for SPWs of both polarities (Fig 1B). However, as not every SPW exhibited a pronounced ripple component (Fig 1A) and ripple events within a single recording displayed variable frequencies, the ripple component was not investigated further. In all cases SPWs were gradually reduced in amplitude and frequency after kainic acid (KA, 400 nM) application (Fig 1A), followed by a brief transitory state with no clear main network pattern. Subsequent gamma network oscillations appeared with a delay and increased progressively in amplitude and power until reaching a steady state (Fig 1A).

**Fig 2:**
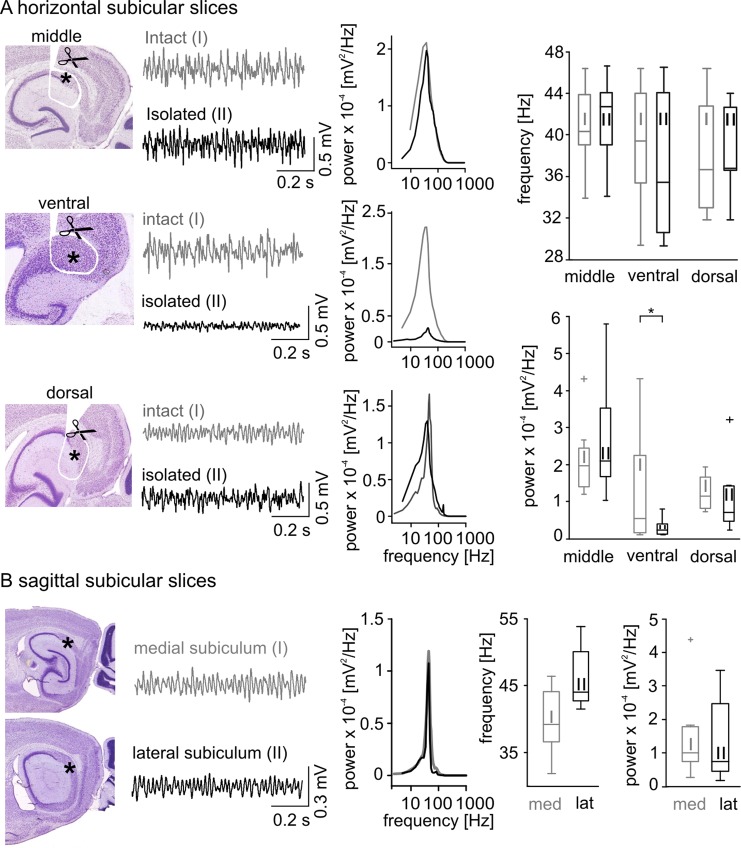
Gamma frequency network oscillations within the subiculum. (A) Gamma frequency oscillations were found in all of the examined subicular regions (middle, ventral, and dorsal). Sketches illustrate horizontal (middle, ventral and dorsal) slice preparation. The position of the scissors indicate the cuts made around the perimeters of the subicular region with the resulting subicular minislices marked by an asterisk. (A, right next to the sketch) Two example LFP recordings obtained from intact (grey, I, top trace) and isolated (black, II, bottom trace) middle (top), ventral (middle) and dorsal (bottom) slices are displayed together with the corresponding power spectra (A, middle column, color code according to the example traces). (A, right) The population data of the oscillatory frequency (top histogram) and spectral power (bottom histogram) exhibits no significant difference of the intact compared to the isolated subicular slices in network oscillatory gamma frequency as well as in spectral power (values and numbers in Table 1) except for the ventral subicular slices (*p* = 0.034, significance level indicated by asterisk). (B) Gamma frequency oscillations recorded from the medial (grey, I) and lateral (black, II) subiculum within the sagittal slice preparations. Same type of illustration as in (*A*), the subicular region is marked by an asterisk. The population histogram for frequency and power (values and numbers in Table 2) did not reveal a significant difference.

In order to evaluate the subicular capability to generate gamma frequency oscillations independently, we compared local network oscillations from 12 intact and 24 isolated subicular horizontal middle slice preparations (Fig 2). In the intact slices, the frequency and power of gamma oscillations were 40.5 ± 3.7 Hz and 2.09 x 10^-4^ ± 8.35 x 10^-5^ mV^2^/Hz, respectively. Minislices containing an isolated subiculum displayed a slightly increased but not significantly different frequency and spectral power (*p* = 0.41 and *p* = 0.19, respectively, Fig 2A, Table 1). Gamma frequency oscillations were also detected in more dorsal and ventral minislices (Fig 2A, Table 1), as well as in medial and lateral sagittal slice preparations (Fig 2B, Table 2), altogether indicating a robust intrinsic subicular gamma generator. These results support the idea that the subiculum is capable of generating gamma network oscillations independent of an external drive.

**Table 1:** Properties of gamma frequency oscillations in the intact and isolated subiculum.

horizontal slices		n	frequency [Hz]	power [V^2^/Hz]
ventral	intact	7	39.5 ± 5.6	1.4 x 10^-4^ ± 1.5 x 10^-4^
	isolated	12	37.1 ± 6.6	3.0 x 10^-5^ ± 2.1 x 10^-5^
middle	intact	12	40.5 ± 3.7	2.1 x 10^-4^ ± 8.4 x 10^-5^
	isolated	24	41.6 ± 3.9	3.0 x 10^-4^ ± 2.2 x 10^-4^
dorsal	intact	4	37.9 ± 5.6	1.3 x 10^-4^ ± 4.7 x 10^-5^
	isolated	7	38.4 ± 4.1	1.2 x 10^-4^ ± 9.5 x 10^-5^

**Table 2:** Properties of gamma frequency oscillations within the subiculum in the sagittal slice preparation.

		n	frequency [Hz]	power [mV^2^/Hz]
sagittal slices	medial	11	40.0 ± 4.7	2.2 x 10^-4^ ± 2.9 x 10^-4^
	lateral	8	44.3 ± 7.8	1.4 x 10^-4^ ± 1.2 x 10^-4^

### Electrophysiological properties of subicular PCs

**Fig 3:**
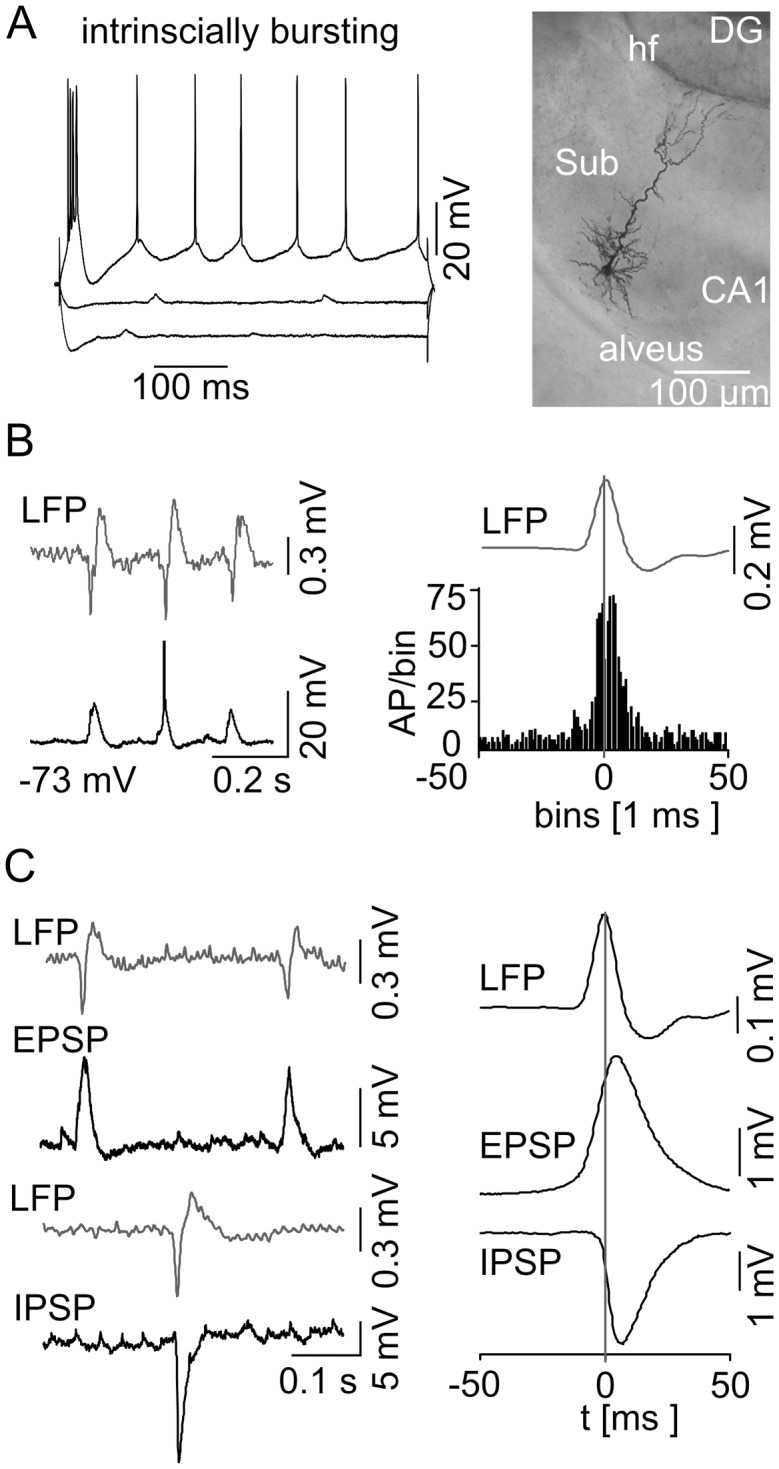
Behavior of subicular IB cells during spontaneous subicular SPWs. (A) Current-voltage relationship of a IB cell (left) with current injection steps of −300 pA, −100 pA, and +140 pA, respectively, displayed with the attached microphotograph of a biocytin-stained IB cell (right). These cells exhibit the typical pyramidal shaped cell body, prominent apical dendrites that travel through the molecular layer reaching the hippocampal fissure (hf), and basal dendrites that spread within the pyramidal cell layer. The axon leaves the subiculum (Sub) via the alveus. IB cells respond to a hyperpolarizing current injection with a sag in membrane potential whereas a positive current pulse leads to burst firing. (B) Example of simultaneous extracellular LFP (top trace) and intracellular (bottom trace) recordings at RMP is shown on the left. The intracellular recording reveals phase-locked synaptic responses as well as a full-blown AP (truncated for clarity) with respect to the LFP SPWs. The spike time histogram (n = 16 IB cells) on the right illustrates a clear peak of AP generation in close vicinity to the SPW peak. The vertical line marks the maximum mean SPW deflection as time point 0. (C) EPSPs and IPSPs are displayed in correlation to the LFP (left). The EPSPs and IPSPs were recorded at −80 mV and at 0 mV, respectively. (right) Accumulated mean EPSP/IPSP with respect to the maximum SPW peak deflection.

**Fig 4:**
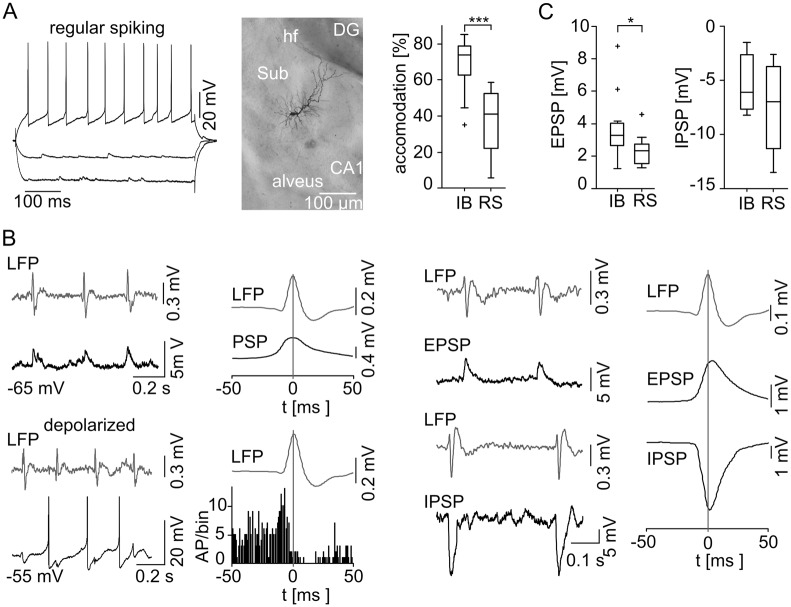
Behavior of subicular RS cells during spontaneous subicular SPW activity. (A) Neuronal discharge pattern and microphotograph of an RS cell. RS cells do not fire bursts and show little or no sag potential. They respond to a depolarizing current injection with a train of single APs. RS cells show a typical pyramidal cell morphology. (A, right) The accommodation behavior (n = 19 IB cells; n = 16 RS cells) reveals a significant difference between subicular IB and RS cells (level of significance indicated by the asterisks, *p* < 0.0001). (B) Example of a RS cell at RMP (top left) and under the condition of depolarizing current injection (bottom left) during spontaneous SPW is given with the same type of illustration as in Fig 3. Intracellular example recording of a silent RS cell at RMP (top left) depicting a SPW associated postsynaptic depolarization without AP generation. The population data (n = 7, right next) do not contain any APs, but aggregate a phase-locked mixed postsynaptic current (PSP) instead. When depolarized by current injection previously silent RS cells display a tonic AP firing mode (bottom left). In stark contrast to the IB cells, data of spontaneous active and depolarized RS cells reveal a prominent SPW peak correlated pause of AP generation (n = 11, bottom, middle left). EPSPs and IPSPs examples are displayed in correlation to the LFP (middle right). The EPSPs and IPSPs were recorded at −80 mV and at 0 mV, respectively. (right) Accumulated mean EPSP/IPSP with respect to the maximum SPW peak deflection. (C) EPSP (left) and IPSP (right) amplitudes for both cell classes. IB cells receive significantly higher synaptic excitation than RS neurons (indicated by the asterisk, *p* = 0.033; IB: n = 12; RS: n = 13), while there was no significant difference in IPSP (*p* = 0.52; IB: n = 3; RS: n = 4).

We performed intracellular recordings from 42 subicular PCs (42 slices) and classified 24 of them (57%) as IB cells based on their ability to fire bursts of APs in response to a moderate depolarization (Fig 3A). The interspike interval within a burst was 5.0 ± 1.1 ms (n = 24, see also [6,16,34]). The initial burst was followed by a train of single APs with only slight accommodation if the depolarization was maintained (Fig 3A, Table 3; [5,44]). 18 from 42 cells (43%) were identified as RS neurons due to the lack of burst firing during the depolarizing test stimuli (Fig 4A). These neurons responded to a moderate depolarizing current injection with a train of APs displaying a prominent adaptation of the AP firing rate. Hence, the accommodation behavior of the two subicular PCs classes differed highly significantly (*p* < 0.0001, Fig 4A, Table 3). There were no significant differences concerning the intrinsic properties and AP amplitude, half-width or firing threshold (Table 3; [34]). However, the AP shapes exhibited a variation between the two cell classes: RS cells displayed a prominent afterhyperpolarization (AHP) whereas IB cells showed this only very infrequent and to a lesser degree (Table 3). An afterdepolarization (ADP) was observed in IB but not RS neurons and vanished when the cells were depolarized. Furthermore, IB cells displayed a hyperpolarization-activated sag potential (Fig 3A, see also [6,9,17,18,21,44]). In RS cells, the sag was not constantly observed and if present, displayed a significant smaller maximum deflection (*p* < 0.005, Table 3). 20 electrophysiologically characterized cells (11 IB and 9 RS) were stained with biocytin. All stained neurons exhibited the typical pyramidal cell shaped soma (Figs 3A and 4A). Dye coupling was found exclusively in one stained IB cell. Hence, subicular IB and RS cells clearly form two separate subicular pyramidal cell classes based on their ability to generate AP bursts upon moderate depolarization, the distinct accommodation behavior and different AP waveform features.

**Table 3:** Electrophysiological properties of subicular PCs.

	IB (n)	RS (n)	*p* value
RMP [mV]	–67.5 ± 7.3 (24)	–70.3 ± 8.4 (18)	0.2630
R_in_ [MΩ]	85.6 ± 37.8 (23)	98.2 ± 37.1 (18)	0.3057
τ [ms]	6.4 ± 3.8 (23)	7.0 ± 2.9 (18)	0.5859
AP amplitude [mV]	67.5 ± 10.8 (24)	68.5 ± 9.5 (18)	0.7607
AP half-width [ms]	0.9 ± 0.2 (24)	1.0 ± 0.2 (18)	0.0776
AP threshold	–58.9 ± 5.7 (23)	–56.2 ± 7.7 (17)	0.2331
ADP [mV]	4.7 ± 4.5 (23)	0.2 ± 0.7 (18)	0.0002 **
AHP [mV]	–1.3 ± 1.7 (24)	–3.8 ± 2.5 (18)	0.0006 **
sag -300pA [%]	14.3 ± 9.0 (22)	6.2 ± 7.1 (18)	0.0045 **
sag -200pA [%]	16.2 ± 10.0 (23)	6.7 ± 7.3 (18)	0.0021 **
sag -100pA [%]	18.5 ± 9.4 (23)	6.7 ± 7.2 (18)	0.0001 **
accommodation [%]	69.1 ± 12.9 (19)	36.7 ± 16.8 (16)	<0.0001 ***

### Subicular PCs behavior during SPW activity

During spontaneously occurring SPW, the majority of IB neurons were active (16/24, 67%) whereas RS cells remained predominately silent (14/18, 78%). The AP generation of the active IB cells depicted a striking phase-locked behavior around the peak of the field SPW (Fig 3B). Most of the silent IB cells displayed a similar firing pattern generating APs upon further depolarization using intracellular current pulses (5 of 8 silent IB cells). Three active and one inactive IB neurons displayed an evident hyperpolarization at the peak of SPW oscillation resulting in a disruption of AP firing. These cells further exhibited a significantly higher τ of 14.0 ± 2.0 ms compared to the phase-locked IB cells (4.8 ± 1.6 ms, n = 16, *p* < 0.0001) together with a significantly less prominent sag potential at −100 and −300 pA (*p* = 0.0012 and *p* = 0.046, respectively). Finally, two silent IB cells were discarded from further analysis due to insufficient numbers of triggered APs in our recording.

At RMP the majority of RS neurons (14/18, 78%) remained ‘silent’ and did not initiate APs during SPW episodes, even though a mixed excitatory and inhibitory postsynaptic potential (EPSP and IPSP, respectively) was apparent (Fig 4B). When depolarized above threshold, those cells started to fire but, in stark contrast to the IB cells, showed a pause of AP firing at the SPW peak (Fig 4B). Three of those cells had to be excluded from further analysis due to an insufficient AP number in our recording. Only one RS cell fired phase-locked to the SPW peak. In contrast to the comparable IPSP amplitudes in both PC classes (IB: −5.3 ± 2.8 mV, n = 3; RS: −7.6 ± 4.2 mV, n = 4; *p* = 0.52), we found a significant larger EPSP in IB compared to RS neurons (IB: 3.7 ± 0.2 mV, n = 12; RS: 2.3 ± 0.1 mV, n = 13; *p* = 0.033; Fig 4C). However, the time point of maximal cumulative EPSP deflection was similar (IB: 5 ms, n = 12, RS: 4 ms, n = 13) whereas the peak of cumulative inhibitory synaptic input was reached 3.5 ms earlier in RS (n = 4, Fig 4B) compared to IB cells (n = 3, Fig 3C). Evaluating the EPSP to IPSP time difference for each neuron separately, a mean time difference of 3.7 ms for the synaptic current relation of both cell classes was gained, once again with a similar mean EPSP peak time in both cell types but an earlier mean IPSP peak time for RS cells.

### Involvement of subicular PCs in gamma frequency oscillations

**Fig 5:**
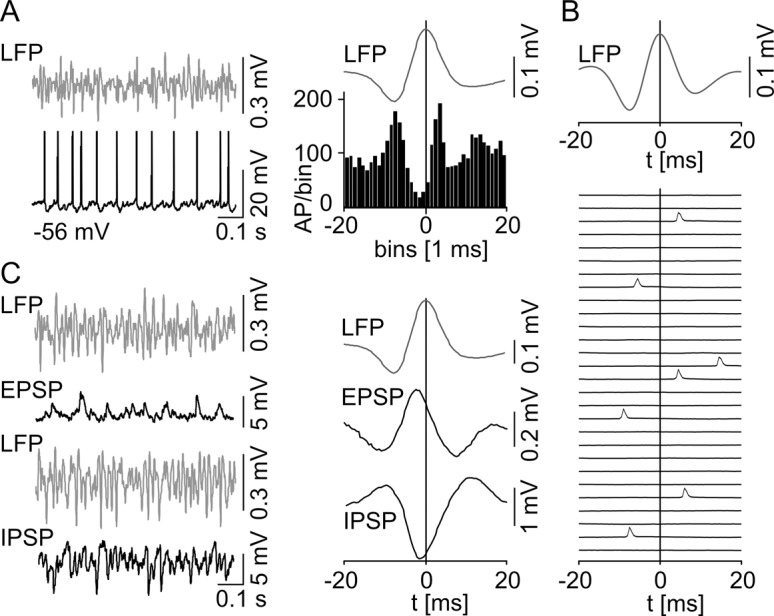
Temporal correlation of subicular IB cell activity to subicular field gamma frequency oscillations. (A) Example of simultaneous extracellular LFP (top trace) and intracellular (bottom trace) recordings demonstrates regular AP firing (truncated for clarity) during gamma network oscillations. The spike time histogram (n = 8) on the right reveals a bimodal phase-locked behavior with a prominent pause of AP generation around the peak of the gamma cycle. The vertical line marks the maximum mean gamma peak deflection as time point 0. (B) Single intracellular recording cutouts (40 ms each) of consecutive triggered LFP gamma cycles illustrating the typical IB cell AP pattern for oscillatory network gamma activity together with the mean LFP trace of these recordings on top. (C) Example traces of synaptic potentials (left). EPSP and IPSP were recorded at −80 mV and 0 mV, respectively. Maximal cumulative postsynaptic potential peak deflections occur before the maximum of LFP gamma cycle (right).

We recorded from a total of 22 subicular PCs during both spontaneous SPW and subsequent gamma frequency network oscillations. 11 (50%) of these cells were identified as IB and 11 (50%) as RS neurons. The majority of IB cells (10/11, 91%) were active with only one cell remaining silent during the gamma rhythm. Within the active gamma network, IB cells displayed a significantly higher depolarization of 9.2 ± 2.8 mV than RS neurons, 6.3 ± 3.0 mV (*p* = 0.037), resulting in a significantly lower membrane potential (Table 4). There was no significant difference in the R_in_ and τ between the two subicular cell classes, suggesting that the observed difference in membrane potential reflects a network induced variation. In the active network, IB cells displayed a reduced sag potential and ADP (Table 4). A hyperpolarizing current injection did not lead to the recovery of the ADP, which suggests a network-induced effect as well. Even when hyperpolarized to the initial RMP, IB cells failed to generate burst activity and continued to fire only single APs in correlation to the active gamma field. The firing pattern of most of the IB cells (8/11, 73%) displayed a bimodal peak of AP discharge in the spike-time histogram with a prominent pause before the peak of gamma frequency oscillations (Fig 5A). These cells discharged with a frequency of 11.4 ± 4.6 Hz (n = 6) and fired only once per gamma cycle. The bimodal peak in the spike-time histogram cannot be explained by spike doublets or rebound depolarization [33]. IB cells generated only one AP per gamma cycle, but continuously switched their activity between different phases of the ongoing gamma network oscillation (Fig 5B). 3/11 IB cells (27%) showed diffuse AP firing with no clear phase correlation to the gamma field. There were no intrinsic electrophysiological difference between these two groups of subicular IB cells that could account for the different firing behavior.

**Table 4:** Electrophysiological properties of subicular PCs during gamma frequency oscillations.

	IB	RS	*p* value
MP [mV]	–59.6 ± 4.5 (11)	–65.6 ± 7.5 (11)	0.0422 *
depolarization [mV]	9.2 ± 2.8 (11)	6.3 ± 3.0 (11)	0.0367 *
R_in_ [MΩ]	99.7 ± 41.6 (11)	84.1 ± 21.2 (11)	0.3033
τ [ms]	6.5 ± 3.6 (11)	6.4 ± 3.4 (11)	0.9680
AP amplitude [mV]	66.9 ± 7.3 (11)	66.2 ± 10.7 (10)	0.8660
AP half-width [ms]	1.0 ± 0.1 (11)	1.0 ± 0.1 (10)	0.6521
AP threshold	–59.4 ± 8.5 (11)	–51.8 ± 9.6 (9)	0.0932
ADP [mV]	1.1 ± 1.9 (11)	0.0 (11)	0.0828
AHP [mV]	–2.0 ± 2.1 (11)	–2.7 ± 2.0 (11)	0.4183
sag -300pA [%]	12.4 ± 6.5 (11)	7.1 ± 8.0 (11)	0.1118
sag -200pA [%]	13.6 ± 5.8 (11)	5.1 ± 7.1 (11)	0.0133 *
sag -100pA [%]	13.7 ± 9.5 (11)	5.2 ± 4.9 (11)	0.0204 *

**Fig 6:**
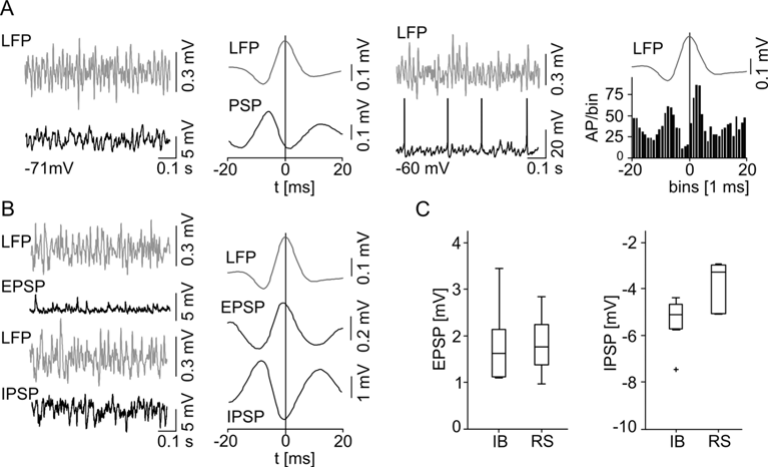
Temporal correlation of subicular RS cell activity to gamma frequency network oscillations. (A) Examples of simultaneous LFP (top) and intracellular (bottom) recordings during gamma frequency oscillations. In stark contrast to IB neurons, without current injection RS cells usually do not generate APs during gamma frequency oscillations and the data (n = 7) solely reveals a phase-locked mixed postsynaptic current (PSP, middle left). A depolarizing current injection initiates AP generation (middle right). Depolarized and spontaneous active RS cells (n = 9) show a distribution of AP generation (right) similar to the one observed in IB cells. (B) Example traces of synaptic potentials on the left. EPSP and IPSP were recorded at −80 mV and 0 mV respectively. Maximal cumulative postsynaptic potential peak deflections occur before the maximum of LFP gamma cycle. (C) There was no significant difference between the two classes of subicular PCs concerning the EPSP (IB: n = 8, RS: n = 11; *p* = 0.86) and IPSP amplitude (IB: n = 5, RS: n = 5; *p* = 0.56).

**Fig 7:**
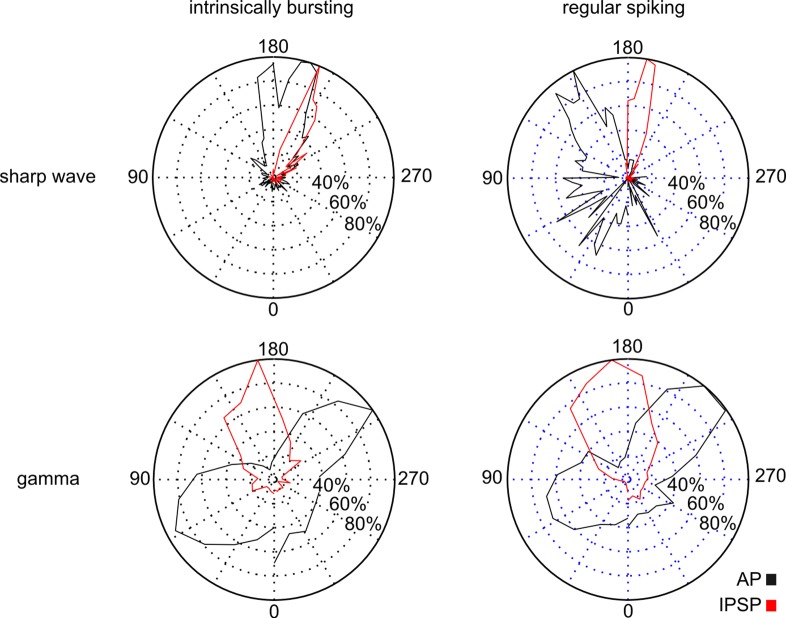
Phase distribution of APs and synaptic inhibition during SPW and gamma frequency oscillations. APs (black) and synaptic inhibition (red) of IB (left column) and RS (right column) cells projected on a standard phase polar diagram for SPW (upper row; 80 ms projected time window) and gamma frequency oscillations (lower row; 24 ms projected time window). The peak of network activity is located at 180°, the ascending slope is encoded with lower, the descending with higher values. APs are illustrated based on the cumulative AP time histograms (Figs 3B, 4B, 5A and 6A) with respect to the projected time window, synaptic inhibition is represented by the distribution of time points for maximal deflection in the cellular cumulative IPSPs (Figs 3C, 4B, 5C and 6B). All values are normalized to the respective maximum activity level. During SPW oscillations RS cells, spontaneously active or depolarized, exhibit an earlier AP activity (n = 11) as well as an earlier peak of synaptic inhibition (n = 4) with respect to the IB cell phase distribution (n = 16, n = 3, respectively). Hence, according to the different phase distributions the activity of both cell types is separated in the time domain during SPW network activity. In contrast, during gamma frequency oscillations both subicular cell classes reveal comparable activity pattern with two distinct AP peaks (IB: n = 8; RS: n = 9) and an intermediate prominent inhibition (IB: n = 5; RS: n = 5).

In marked contrast, the majority of RS cells (7/11, 64%) remained silent during the gamma network rhythm. Interestingly, two neurons that were inactive during the SPW changed activity mode and became active during gamma frequency oscillations. The silent RS cells displayed a mixed EPSP/IPSP (Fig 6A). When depolarized above threshold, however, it was remarkable that similar to the IB cells, 8/11 RS neurons showed AP firing with a pause of discharge before the peak of gamma frequency oscillations. Again, there was no indication for doublets or rebound depolarization. In addition, we analyzed the inter-spike-interval (ISI) for all recorded APs of both cell types during the gamma rhythm. In RS cells, we found no ISI shorter than 20 ms and for IB cells, only 0.91% of all analyzed ISI were below 20 ms, altogether supporting the conclusion that spike doublets or rebound spiking are not prevalent during ongoing oscillatory gamma network activity. The EPSP amplitude of IB cells was 1.79 ± 0.8 mV (n = 8) and the IPSP amplitude was −5.36 ± 1.1 mV (n = 5) resulting in an EPSP/IPSP ratio of 0.33. RS cells displayed an EPSP and IPSP amplitude of 1.84 ± 0.6 mV (n = 11) and −4.48 ± 2.6 mV (n = 5), respectively, with an EPSP/IPSP ratio of 0.41. There was no significant difference in the amplitude of synaptic inputs (EPSP: *p* = 0.86; IPSP: *p* = 0.55, Fig 6C). The peak amplitude of cumulative excitatory and inhibitory synaptic inputs for IB (time to LFP peak: EPSP −1.5 ms; IPSP 1.5 ms, Fig 5C) and RS (time to LFP peak: EPSP −1 ms; IPSP −0.5 ms, Fig 6B) cells were phase-correlated to the peak of the underlying gamma oscillations. The previous described dichotomy of both cell classes, active IB and ‘silent’ RS cells, was confirmed during gamma network oscillations suggesting a general pattern. However, in contrast to the SPW, both cell types predominantly exhibited comparable AP pattern and phase-locked behavior during gamma frequency oscillations, which is depicted by the phase distribution of the synaptic inhibition and AP generation (Fig 7). Those phase distributions exhibit a similar profile for both cell types during oscillatory gamma network activity, but an earlier IPSP as well as AP peak time for RS in comparison to IB cells during SPW.

## Discussion

We studied the participation of subicular IB and RS neurons in two major network rhythms, SPW and gamma frequency oscillations. The majority of IB cells were active during both network states whereas the RS cells remained predominantly silent. Due to the ability of the isolated subiculum to generate network oscillations independently, we conclude that the activation of IB cells is sufficient to drive the rhythmic activity within the subiculum. For IB cells a low or moderate network activation, like the one observed in this study, could represent a sufficient excitatory drive for firing while, in marked contrast, RS cells seems to need a high network excitation or additional excitatory inputs in order to participate in an independent and specific manner in the generation of oscillatory activity. Hence, the distinct activation condition of the two subicular PC classes suggests a bimodal working model operating under different levels of excitation. Furthermore, the disparate activation level together with the distinct phase relation during SPW indicate that IB and RS cells might be involved in different information processing streams. Consequently, our data support the idea of distinct processing channels within the subiculum.

### Two distinct classes of subicular PCs

The recorded 42 subicular PCs were classified as IB (57%) and RS (44%) cells. According to earlier studies, the two subicular cell classes constitute a homogenous group concerning their intrinsic properties [10,16,34] with similar RMP, R_in_, and τ. Nevertheless, in addition to the fundamental eponymous ability to generate bursts of APs under moderate current injection, we found, in line with other reports, distinct characteristics in active membrane properties like the sag potential and AHP of subicular PCs (Table 3 and Table 4, [8,9,21,35]). However, none of these physiological parameters were exclusive for one cell type and therefore not sufficient for a distinguished categorization. The very prominent disparate feature of the accommodation behavior might, in this context, be an exception and could therefore serve as an adequate indicator for the correct classification.

### SPW and gamma oscillations are competitive rhythms within the subiculum

Using slice preparation, we found, in line with an earlier report [14], that *in vitro* spontaneous SPWs are present in the subiculum. We further confirmed the occurrence of two SPW polarities [14, 36], both of them exhibiting superimposed fast ripple oscillations in a more or less pronounced manner. The occurrence of subicular SPW polarity could dependent on the location within the subiculum [14] or their different generation mechanism [36]. The exact mechanism, however, remains unclear demanding further investigations. Nevertheless, analyses of our data with respect to the SPW polarity did not reveal a statistical significant difference.

In addition, we were able to demonstrate that the subiculum constitutes a stable gamma generator following subicular isolation in acute slice preparation. SPW and gamma frequency oscillations that are generated during different behavioral states in freely moving animals [37] are competitive rhythms within the subiculum *in vitro*, possibly suggesting the representation of state-dependent information processing based on the involvement of similar cell assemblies.

### Participation of IB and RS cells in SPW and gamma frequency network oscillations

The two pyramidal cell classes showed a fundamentally different activity level: while most of IB cells were active during SPW and gamma frequency oscillations, the majority of RS cells remained ‘silent’ without AP generation. RS cells, even though a mixed postsynaptic current was recorded, essentially needed a further depolarization in order to switch into the participating mode. The neural activity of IB cells displayed, in contrast to a recent report [38], a striking phase coupling to the peak of SPW (see also [14]). During the SPW rhythm, the majority of IB cells were active with a pronounced excitatory input, resulting in the generation of APs in obvious correlation to the peak of SPW deflection. In marked contrast, when depolarized above threshold, RS neurons showed AP firing with a prominent inhibition following the peak of the SPW network rhythm. This peak of aggregated IPSPs occurred earlier than in IB cells, parallel to the earlier peak in the spike time histogram (Fig 7), altogether suggesting that RS cells receive a different set of synaptic inputs. Sharp wave ripples are supposed to represent behavioral relevant activation of neuronal cell assemblies in a time compressed manner [39]. Hence, a distinct neuronal activity pattern segregated in the time domain of SPW rhythms is an important clue for a task related differentiation of IB and RS cells.

The functional dichotomy of IB and RS cells was retained during subsequent gamma frequency oscillations. The pronounced excitability of IB cells here cannot be attributed to the diverse strength of synaptic inputs because both cells exhibited comparable EPSPs and IPSPs (Fig 5 and Fig 6). It rather seems to result from a network-triggered activation of an intrinsic neuronal process which stresses the functional dichotomy of subicular PCs. Corresponding to the increased excitability in IB cells, we found a stronger network oscillation associated depolarization of the membrane potential. In support of this, the application of the cholinergic agonist carbachol, in a concentration sufficient to induce gamma oscillations, leads to a depolarization in subicular IB cells [40,41]. However, IB and RS cells exhibited a comparable phase distribution with a bimodal phase locking behavior (Fig 7). During both network states inhibition essentially shaped the output pattern (Fig 7), which is well in line with previous reports [13,42]. The loss of GABAergic inhibition within the subiculum has been associated with the uncontrolled discharge in temporal lobe epilepsy [43,44].

Hence, network oscillations observed under our condition are mainly based on the IB cell activity while RS neurons can potentially participate in the rhythm generation in a specific and independent manner if further depolarized. Consequently, the subiculum seems to operate in two different working modes: under the regime of low or moderate excitability, like the one observed in this study, RS cells generally remain silent, while under conditions of sufficient excitatory input RS cells can participate in oscillatory network activity. This additional network drive might stem from a higher general excitation of the subiculum via specific additional excitatory inputs independent from IB cells. In line with our suggestion, within a complete hippocampal preparation theta-coupled spontaneous slow and fast gamma activity can be detected in the subiculum with phase-coherent RS activity [29]. Furthermore, not only IB but also RS cells are involved in population activity of a subicular *in vitro* epilepsy model, indeed with a clear leader function of the IB cell type [13]. However, the conditions for recruitment of potentially participating RS cells need further investigation.

### Information processing within the subiculum

Based on the distinct target areas, firing properties, electrophysiological features, pharmacological modulation, and long-term potentiation [17–21], it has been assumed that IB and RS cells constitute parallel pathways presumably processing distinct modalities of information. In addition, our data indicate that both cell types are differentially recruited in the active network, thus supporting the idea of different information processing channels. However, in parallel to the non-exclusive physiological membrane characteristics in each PC class, some neurons exhibited divergent oscillatory AP pattern with respect to the main classification scheme. It is important to note that the statistical correlation of subicular afferent and efferent connectivity [45] is not completely revealing, especially for medial slices with a mixture of 50% IB and RS cells, overall emphasizing a predominant but not exclusive anatomical projection pattern. The aim of neuronal classifications is to gain a deeper understanding of the fundamental circuitries and the corresponding information processing streams. But as recently demonstrated for parvalbumin-positive basket cells [46] even a homogenous considered group of neurons can functionally constitute local heterogeneous microcircuits. Consequently, we have to admit that information processing in the subiculum seems to be more diverse than initially assumed, possibly based on the recruitment of different cell types in a task specific manner. The dissection of functional processing modes therefore needs further investigation in order to gain a fine scale typology. We believe that *in vitro* models permitting the investigation of different network pattern on a cellular level, like the one used here, will be a valuable tools for such purposes.
